# Survey of Obstetrics and Gynecology Residents Regarding Pneumococcal Vaccination in Pregnancy: Education, Knowledge, and Barriers to Vaccination

**DOI:** 10.1155/2016/1752379

**Published:** 2016-02-02

**Authors:** Emily E. Fay, Kara K. Hoppe, Jay Schulkin, Linda O. Eckert

**Affiliations:** ^1^Department of Obstetrics and Gynecology, University of Washington School of Medicine, Seattle, WA 98195, USA; ^2^Department of Obstetrics and Gynecology, University of Wisconsin School of Medicine and Public Health, Madison, WI 53726, USA; ^3^Department of Research, American College of Obstetricians and Gynecologists, Washington, DC 20024, USA; ^4^Department of Global Health, University of Washington School of Medicine, Seattle, WA 981985, USA

## Abstract

*Objective.* The 23-valent pneumococcal vaccine is recommended for adults over 65 years of age and younger adults with certain medical conditions. The Centers for Disease Control and Prevention (CDC) state insufficient evidence to recommend routine pneumococcal vaccination during pregnancy, but the vaccine is indicated for pregnant women with certain medical conditions. We designed this project to gauge obstetrics and gynecology (OB/GYN) resident knowledge of maternal pneumococcal vaccination.* Methods.* We administered a 22-question survey to OB/GYN residents about maternal pneumococcal vaccination. We performed descriptive analysis for each question.* Results.* 238 OB/GYN residents responded. Overall, 69.3% of residents reported receiving vaccination education and 86.0% reported having ready access to vaccine guidelines and safety data. Most residents knew that asplenia (78.2%), pulmonary disease (77.3%), and HIV/AIDS (69.4%) are indications for vaccination but less knew that cardiovascular disease (45.0%), diabetes (35.8%), asthma (42.8%), nephrotic syndrome (19.7%), and renal failure (33.6%) are also indications for vaccination.* Conclusion*. OB/GYN residents are taught about vaccines and have ready access to vaccine guidelines and safety data. However, knowledge of indications for pneumococcal vaccination in pregnancy is lacking. Likely, the opportunity to vaccinate at-risk pregnant patients is being missed.

## 1. Introduction

Invasive disease from* Streptococcus pneumoniae* is a major cause of illness, including pneumonia, bacteremia, meningitis, and otitis media [1]. The 23-valent pneumococcal polysaccharide vaccine (PPSV23) is recommended in all adults aged 65 years and older and is recommended in those adults younger than 65 years with certain medical conditions displayed in the list below [2]. The Centers for Disease Control and Prevention (CDC) state insufficient evidence to recommend routine pneumococcal vaccination during pregnancy, but the PPSV23 is indicated for pregnant women with the medical conditions listed in the list below [3, 4]. Given the increasing incidence of obesity and related chronic conditions in the United States, there is likely a significant number of women who meet criteria to receive the pneumococcal vaccine during pregnancy [4]. For these women, the goal of maternal pneumococcal vaccination is both to prevent disease in the mother and also to provide passive immunization to the neonates.

*Indications for pneumococcal vaccination in adults younger than 65 years old *are as follows* (adapted from the Centers for Disease Control and Prevention, pneumococcal disease; available at* http://www.cdc.gov/pneumococcal/index.Html *and retrieved on August 2, 2015)*

- chronic illness: lung, heart, liver, or kidney disease; asthma; diabetes; alcoholism;
- conditions that weaken the immune system: HIV/AIDS, cancer, and damaged/absent spleen;
- living in nursing homes or other long-term care facilities;
- those with cochlear implants or cerebrospinal fluid (CSF) leaks;
- smokers.

Obstetrician-Gynecologists (OB/GYNs) provide more general medical care to women than other primary care providers [5] and therefore play an important role in maternal vaccination. Pregnancy offers a unique opportunity to vaccinate at-risk women, as this may represent the only time some women have access to care. Although a survey of OB/GYNs found that most providers administer some vaccines [6, 7], many barriers exist for immunization, especially in pregnancy. Therefore, many women who should receive vaccines may be missed.

OB/GYN residents are the next generation of obstetric providers. Therefore, assessing their knowledge and practice patterns is important to improve training and enhance vaccination in pregnancy. This project was designed to gauge OB/GYN resident knowledge regarding pneumococcal vaccination during pregnancy, a subject matter not previously studied.

## 2. Materials and Methods

This project was granted an exempt status by the Institutional Review Board of the University of Washington. A 22-question electronic survey was created with questions about pneumococcal vaccination, as well as topics including demographic characteristics, knowledge regarding specific vaccines, opinions of immunization education, and which vaccines are safe to administer during pregnancy. Obstetrics and gynecology residency program directors and department chairs of Accreditation Council for Graduate Medical Education (ACGME) accredited OB/GYN residency programs in the United States were emailed in December 2013 and January 2014 and asked for their participation in the study. Specifically, the program directors and department chairs were asked to forward the email with attached survey to the OB/GYN residents at their institution. Two subsequent reminder emails were sent out. Residents were alerted that the survey was anonymous and voluntary. Data analysis was performed using STATA version 12 (StataCorp, College Station TX). Descriptive statistics were computed for primary analysis. Additionally, responses to knowledge questions were compared between the groups who received didactics versus those who did not receive didactics using chi-square tests. Significance was evaluated at *p* < 0.05.

## 3. Results

The department chairs and program directors for 237 OB/GYN residency programs across the United States were contacted via email. Using the published 2013-2014 ACGME data resource book [8], there were 5021 OB/GYN residents during this time period. However, this number includes OB/GYN residents in residency programs in Puerto Rico, who were not surveyed in this study, so this number actually reflects more residents than were surveyed. Overall, 238 OB/GYN residents responded to the survey. Assuming that every program director and chair forwarded the email to all the OB/GYN residents at his or her program, this gives a response rate of less than five percent.

### 3.1. Demographic and Residency Program Information

As shown in Table 1, respondents included 203 (85.7%) females and 34 (14.4%) males, with 189 (80.8%) identifying as Non-Hispanic white, 18 (7.7%) as Hispanic, nine (3.9%) as African American, and 16 (6.8%) as Asian/Pacific Islander (Table 1). National data on OB/GYN resident demographics finds that 81.0% are female, with 54.4% identifying as Non-Hispanic white, six and four-tenth percent as Hispanic, nine and one-tenth percent as African American, and 11.3% as Asian/Pacific Islander [8]. Residency programs were located in the following time zones, eastern 134 (56.5%), central 61 (25.7%), mountain 11 (4.6%), and pacific which includes Alaska and Hawaii 31 (13.1%), and in the following areas, urban, inner city 113 (47.7%), urban, noninner city 80 (33.8%), suburban 35 (14.8%), rural seven (3.0%), and other two (0.8%).

### 3.2. Training and Education

The majority of residents (69.3%) reported that their residency program provides didactics or training about vaccines and 86.0% reported having ready access to vaccine guidelines and safety data. Residents most often accessed immunization information from the CDC (92.7%), the American College of Obstetricians and Gynecologists (ACOG) (87.2%), and the World Health Organization (WHO) (24.8%) and stated satisfaction with these organizations (for CDC 96.7%, ACOG 92.5%, and WHO 94.9%).

### 3.3. Knowledge

**Figure 1:**
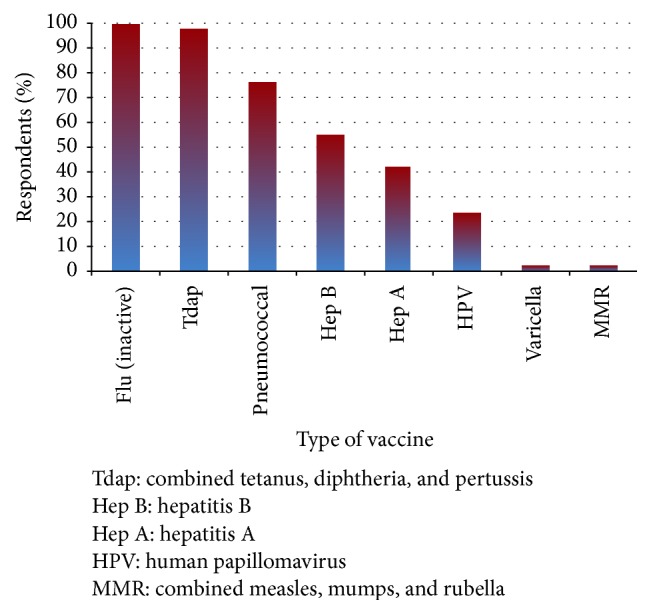
Responses to knowledge questions regarding safe vaccines in pregnancy.

Residents were asked “I think the below vaccines are safe to administer to pregnant patients (check all that apply).” Most residents correctly recognized the combined tetanus, diphtheria, and pertussis (Tdap) (97.5%) and inactivated influenza (99.6%) vaccines as safe and 76.1% of residents identified the pneumococcal vaccine as safe (Figure 1). Only a small percentage incorrectly stated that the measles, mumps, and rubella (MMR) (2.1%) and varicella (2.1%) vaccines were safe in pregnancy (Figure 1). The total number of correct responses was tabulated for each respondent. All respondents had at least four correct responses. This data was further grouped into those with a low number of correct responses, defined as four to six correct responses (68.1% of respondents), or high number of correct responses, defined as seven to nine correct responses (31.9% of respondents). Next, the data was stratified by whether residents received didactics or not. For those residents who reported receiving didactics, there was a nonsignificant trend to having a high number of correct responses (35.8% versus 23.4%, *p* = 0.057), compared to those residents who did not have didactics.

**Figure 2:**
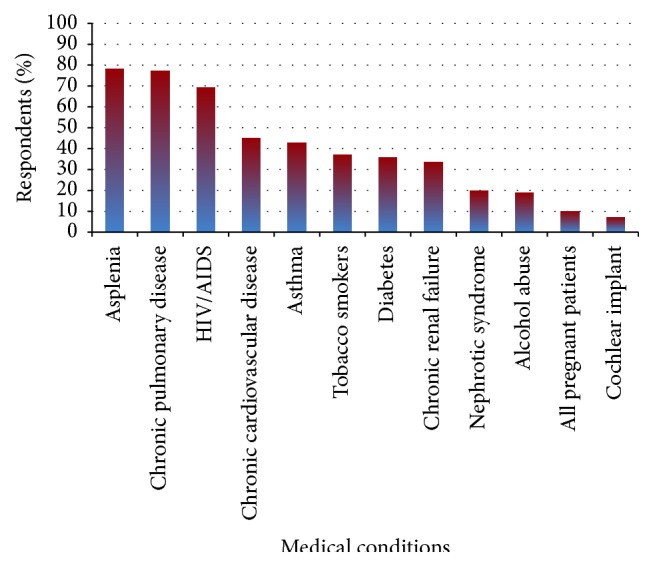
Responses to knowledge questions regarding indications for pneumococcal vaccination in pregnancy.

Residents were then asked to identify indications for pneumococcal vaccination in pregnancy. The majority of residents knew that asplenia (78.2%), chronic pulmonary disease (77.3%), and HIV/AIDS (69.4%) are indications for vaccination but less knew that chronic cardiovascular disease (45.0%), diabetes (35.8%), asthma (42.8%), nephrotic syndrome (19.7%), and chronic renal failure (33.6%) are also indications for vaccination (Figure 2). The total number of correct responses was tabulated for each respondent. This data was further grouped into those with low number of correct responses, defined as zero to four correct responses (38.9% of respondents), moderate number of correct responses, defined as five to eight correct responses (45.0% of respondents), or high number of correct responses, defined as nine to twelve correct responses (16.1% of respondents). Next, the data was stratified by whether residents received didactics or not. For residents who reported receiving didactics there was no significant difference in low, moderate, or high number of correct responses, compared to those who did not have didactics (low: 39.3% versus 38.1%; moderate: 41.8% versus 52.1%; high: 19.0% versus 9.9%, *p* = 0.157).

Additionally, 66% of residents recognized that immunity is provided to the fetus, but only 44.8% recognized that the 23-valent pneumococcal polysaccharide protein conjugate vaccine is the correct one to give in pregnancy to at-risk women.

### 3.4. Barriers

Residents were asked “If you do not offer the pneumococcal vaccine to pregnant patients, please rank the following reasons why you do not.” Answer choices included safety, efficacy, financial reasons/poor reimbursement, not my usual practice, uncertainty regarding recommendations for who should receive the vaccine, availability of the vaccine, perceived unwillingness of patients to accept the vaccine, and other. Seventy-seven (38.1%) reported “not my usual practice” as their number one or two response, and 73 reported (36.1%) “uncertainty regarding the recommendations for who should receive the vaccine” as their number one or two response.

### 3.5. Practices

Residents were asked about their own immunization practices and clinic policies for pneumococcal vaccination in pregnancy. Most residents (96.6%) immunize their pregnant patients. Programs or policies in clinic in place for pneumococcal vaccination include computerized reminder systems (35.3%), regular or ongoing education of healthcare workers about vaccinations (20.6%), reminder notes in patient charts (18.6%), standing orders (15.7%), regular assessment of provider immunization rates (9.8%), and reminder notes in patient rooms or waiting rooms (5.9%). Of the 32 respondents who answered other to this question (31.4%) many of the responses included having no policy in place (17 of 32, 53.1%) or being unaware of any polices (8 of 32, 78.1%).

## 4. Discussion

The goal of maternal vaccination, including pneumococcal vaccination, is twofold: to prevent disease in mothers and to provide passive immunization to neonates. Several studies have examined maternal pneumococcal vaccination, comparing women who received the pneumococcal vaccine during pregnancy to women who received no vaccine or received a different vaccine. These studies found that women who received the pneumococcal vaccine during pregnancy had higher pneumococcal antibodies in their serum [9–13] and breast milk and/or colostrum [10, 14, 15] and in the serum [9–11] and cord blood [12–14] of their infants. While this suggests that vaccination increases both maternal and infant antibody levels to pneumococcus and provides additional antibodies in breast milk, the studies did not assess the clinical impact of maternal vaccination for infants.

A review about the safety of maternal pneumococcal vaccination found no differences in stillbirth [9, 16], spontaneous abortions [16], congenital birth defects [16], or prematurity rates [17] in women who were vaccinated compared to those who were not [18]. These studies only found the expected effects including local tenderness or pain [10, 13, 14, 17], swelling [10, 13, 14], or fever [10, 13, 14, 17]. Hence, all current studies support the safety of maternal pneumococcal vaccination.

Although the pneumococcal vaccine appears to be safe and to benefit both mother and infant, barriers to immunization exist. Most research about beliefs and practices surrounding maternal immunization comes from studies about maternal influenza vaccination. Reported patient barriers to influenza vaccination in pregnancy include safety concerns [19–21], fear of birth defects [22], lack of knowledge about influenza, mistrust of the medical establishment, view of obstetricians as vaccinators, and problems with access to care [19–21, 23]. Although maternal influenza vaccination rates have improved since the 2009 H1N1 influenza pandemic, with coverage now ranging from 32 to 76% nationwide [24], it is still suboptimal.

Provider recommendation for vaccination is important to counter these concerns. Surveys find that 56–89% of women would have received the influenza vaccine during pregnancy if their provider had recommended it [21, 25], and, in a CDC survey, women offered vaccination by their health care provider were five times more likely to have been vaccinated than those who were not offered the vaccine [26].

Barriers to immunization exist among providers as well. Physicians report not providing the influenza vaccine in pregnancy due to lack of safety and efficacy data, concerns about medical legal risks, poor reimbursement, feeling that vaccination is not their scope of practice, and lack of time to inform patients of risks and benefits [7, 27, 28]. Finally, misconceptions of vaccination can also contribute to lower provider administration.

Our data suggest that OB/GYN residents encounter similar barriers to immunization. Many residents expressed that uncertainty of the recommendations for pneumococcal vaccination is one of the top reasons they do not vaccinate their pregnant patients against pneumococcus. Although the majority of residents receive vaccination education and use and are satisfied with other information sources, many lack knowledge regarding pneumococcal vaccination in pregnancy, including the indications for vaccination and the correct vaccine to administer. Residents who receive training about vaccination, compared to those that do not, have a nonsignificant trend towards greater knowledge about safe vaccines in pregnancy, suggesting that didactics bolster knowledge, which may increase the willingness of residents to administer the vaccine to their patients. Furthermore, many residents felt that pneumococcal vaccination was not their usual practice; therefore, many patients who would benefit from this vaccine are likely being missed.

There are multiple limitations to this study. First, because residents were unable to be contacted directly, we asked program directors and chairs to forward the survey, likely limiting the number of residents who received the survey and making it impossible to calculate a true response rate. If every resident received the survey, then our response rate is very low, and therefore no firm conclusions can be ascertained. Moreover, this survey may be limited by self-selection bias. Respondents may have had particular interest in the subject, or other motivation for completing the survey, and therefore may not be a representative sample. Furthermore, since the pneumococcal vaccine is currently recommended only for high-risk patients, residents in a general OB/GYN clinic may not care for the high-risk population who would benefit from this vaccine. A better group to survey may be maternal-fetal medicine fellows and practitioners, who regularly work with pregnant patients for whom this vaccine is recommended.

## 5. Conclusions

Despite the limitations, this survey provokes the question of whether our OB/GYN residents are receiving the correct information about pneumococcal vaccination in pregnancy. Our results suggest that OB/GYN residents are taught about vaccines and have ready access to vaccine guidelines and safety data. However, knowledge of indications for pneumococcal vaccination in pregnancy is lacking. Likely, the opportunity to vaccinate at-risk pregnant patients is being missed. Future studies should investigate this further, and greater work needs to be placed on educating our residents and other obstetric providers about maternal pneumococcal vaccination so that we can ensure the best care for our patients.

## Figures and Tables

**Table 1:** Demographic and residency program information of respondents, year 2014 (*N* = 238).

Characteristic	Number (percentage)
Gender	
Male	34 (14.4)
Female	203 (85.7)
Race/ethnicity (may select multiple answers)	
Non-Hispanic white	189 (80.8)
Hispanic	18 (7.7)
African American	9 (3.9)
Asian/Pacific Islander	16 (6.8)
Native American	1 (0.4)
Multiracial	7 (3.0)
Other	6 (2.6)
Time zone of residency	
Eastern	134 (56.5)
Central	61 (25.7)
Mountain	11 (4.6)
Pacific (including Alaska and Hawaii)	31 (13.0)
Location of residency	
Urban, inner city	113 (47.7)
Urban, noninner city	80 (33.8)
Suburban	35 (14.8)
Rural	7 (2.9)
Other	2 (0.8)

Note: columns may not add up to 100 due to missing data and multiple responses.
